# Supplementary material for: A conserved regulatory program initiates lateral plate mesoderm emergence across chordates
Source: Nat Commun. 2019 Aug 26;10:3857. doi: 10.1038/s41467-019-11561-7 (PMC6710290; doi:10.1038/s41467-019-11561-7)
Supplement: Supplementary file 1 — Supplementary Information [file 41467_2019_11561_MOESM1_ESM.pdf]

Supplementary Information

A conserved regulatory program initiates lateral plate mesoderm emergence across chordates

Prummel, Hess, et al.

Supplementary Figures 1-10
Supplementary Methods
Supplementary Table 1
Supplementary References

Supplementary Figure 1

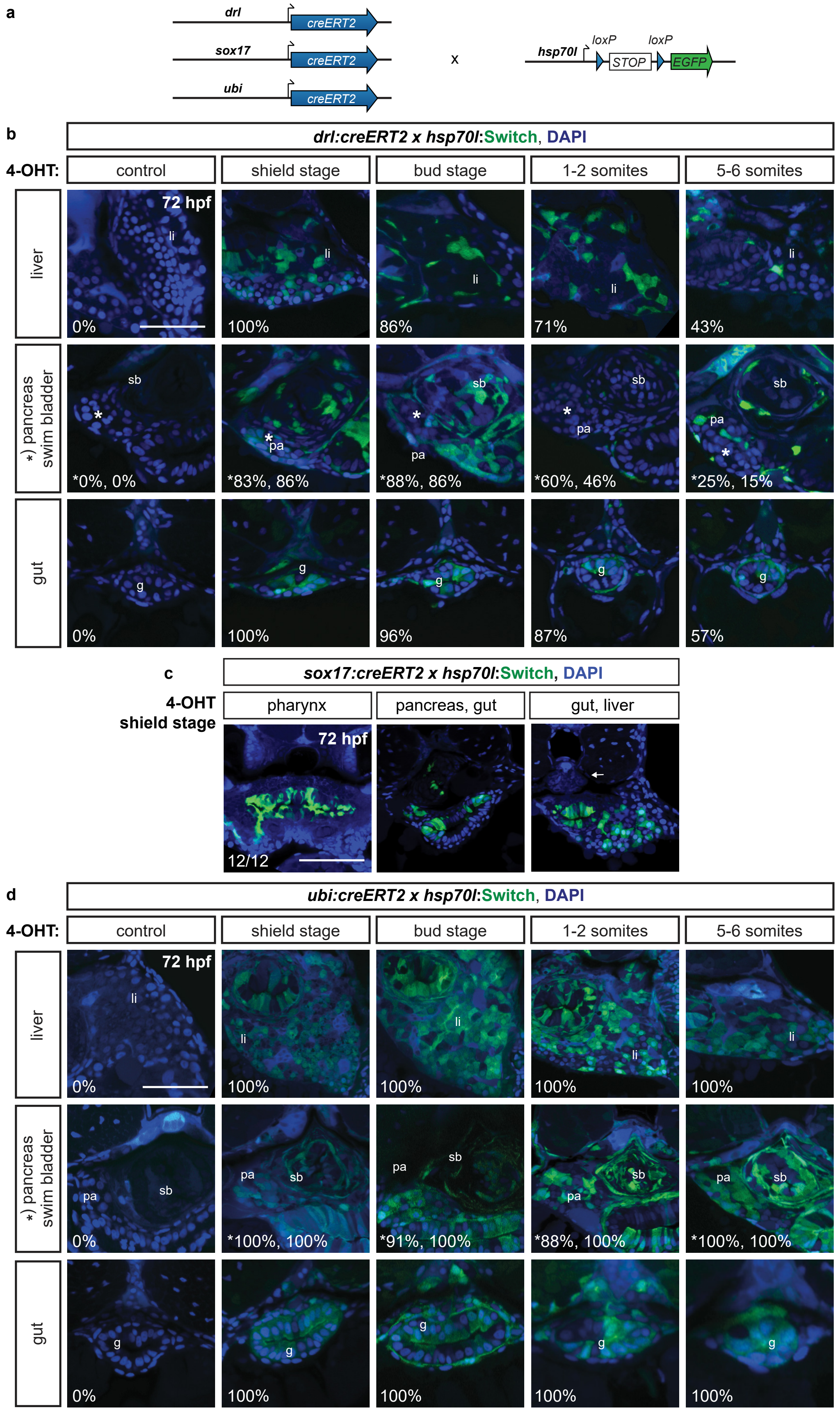

Supplementary Figure 1: *drl* reporter-expressing cells contribute to mesendoderm with LPM-restricted mesoderm contribution
(**a**) Schematic representation of *drl:creERT2*, *sox17:creERT2*, and *ubi:creERT2* crosses to *hsp70l:Switch* for genetic lineage tracing. (**b-d**) Whole-body representative transverse sections of lineage traced embryos at 72 hpf following different 4-OHT induction time points (shield, tailbud, 1-2 ss, and 5-6 ss) to trigger *hsp70l:Switch* recombination (green), nuclei counterstained with DAPI (blue). Numbers indicate the percentage of embryos showing lineage-labeling in depicted organs. (**b**) *drl:creERT2* with 4-OHT induction at shield stage traces, besides of LPM-derived tissue, a high percentage of endoderm-derived lineages, as depicted for liver (li), swim bladder (sb), pancreas (pa), and gut (g), while 4-OHT induction at 5-6 ss traces shows reduced to minimal lineage labeling in endoderm-derived tissue. (**c**) Transverse sections of *sox17:creERT2* lineage-traced endoderm cells after 4-OHT induction at shield stage. Lineage labeling is confined to endodermal lineages and absent from mesodermal and specifically LPM lineages, as depicted for the pharynx, pancreas, gut, and liver, in contrast to LPM-derived tissue such as the aorta (arrow). (**d**) No tissue bias or obvious absence of EGFP in lineage reporter labeling could be detected for the *hsp70l:Switch* line, as shown using the ubiquitous *ubi:creERT2*. Scale bar 50 µm.

Supplementary Figure 2

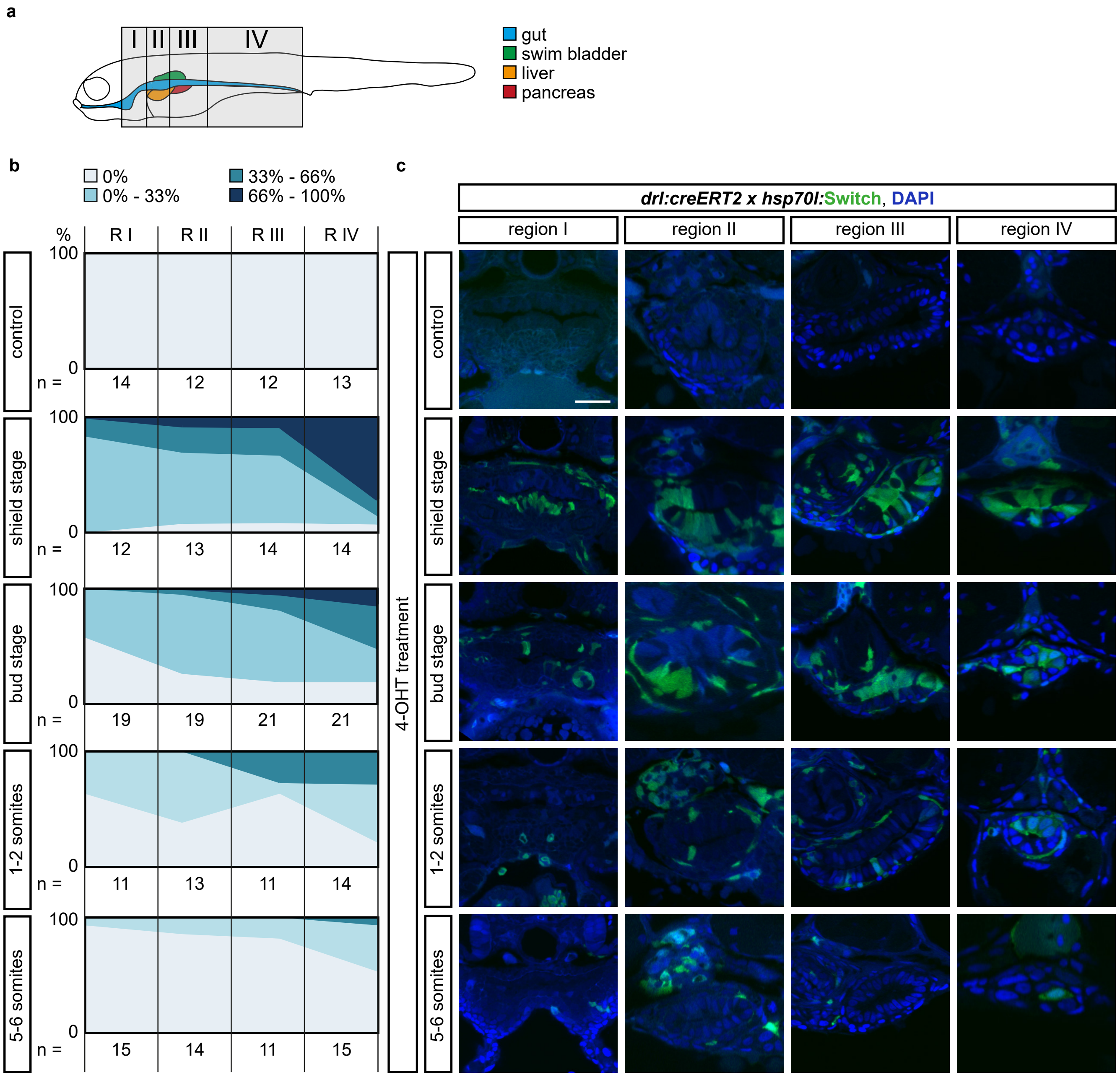

Supplementary Figure 2: The LPM specifies within the mesendoderm with a temporal and an anterior-posterior gradient

(**a**) Schematic of the four regions I-IV defined to study anterior-posterior differences in lineage-labeling of the gut epithelium. Region I is the most rostral and includes the pharynx and the heart; Region II inlcudes the esophagus, the beginning of the swim bladder (pneumatic duct), the liver, and the anterior tip of the pancreas; Region III includes the gut, pancreas, and swim bladder; region IV includes the most caudal part of the gut and is recognizable by the yolk extension. (**b**) Transverse sections of the gut of 3 dpf *drl:creERT2;hsp70l:Switch* embryos. Columns represent the region and rows represent the stage of 4-OHT induction. (**C**) For each section, the switching efficiency in the gut epithelium was classified into 0%, between 0-33%, between 33-66% or between 66-100%. The Y-axis represents the fraction number of embryos. Indicated n-numbers represent the number of embryos analyzed. Embryos were collected in three independent experiments. Scale bar 25 µm.

Supplementary Figure 3

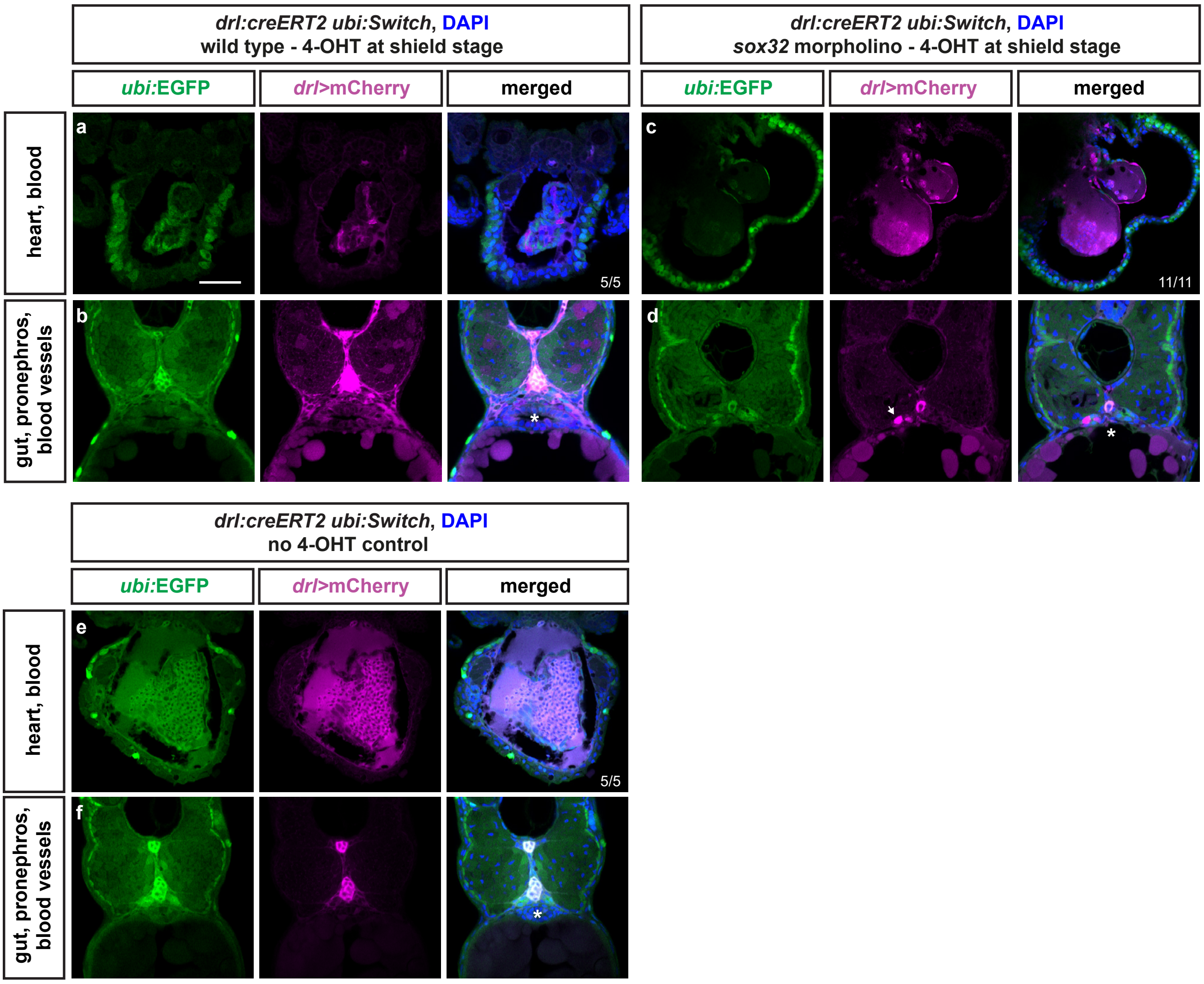

Supplementary Figure 3: LPM lineages and organs still arise in embryos devoid of endoderm

Representative transverse sections of *drl:creERT2* x *ubi:Switch* (*ubi:lox-GFP-lox_mCherry*) embryos fixed at 3 dpf. (**a**,**b**) Embryos were induced with 4-OHT at shield stage, bringing *mCherry* under control of the *ubi* promoter in cells with active CreERT2 from shield stage (magenta), while unrecombined cells keep expressing GFP (green). LPM-derived organs, as depicted for heart, blood, pectoral fins, pronephros, and endothelial cells, and endoderm-derived lineages, including swim bladder, gut epithelium, and liver, are lineage-traced (n=5/5). (**c**,**d**) In *sox32* morphants that are devoid of endoderm, 4-OHT induction at shield stage still traced LPM-derived organs, as depicted for heart, blood, pectoral fins, pronephros (arrow), and endothelial cells (n=11/11). (**e**,**f**) Control without 4-OHT admission reveals absence of any background activity of the used *ubi:Switch* reporter (note autofluoresence of blood), confirming absence of leakiness of the *creERT* and *loxP* lines used (n=5/5). Asterisks indicate endoderm-derived gut epithelium (**b**,**f**) or absence thereof (**d**). Nuclei counterstained with DAPI (blue). Scale bar 25 µm.

Supplementary Figure 4

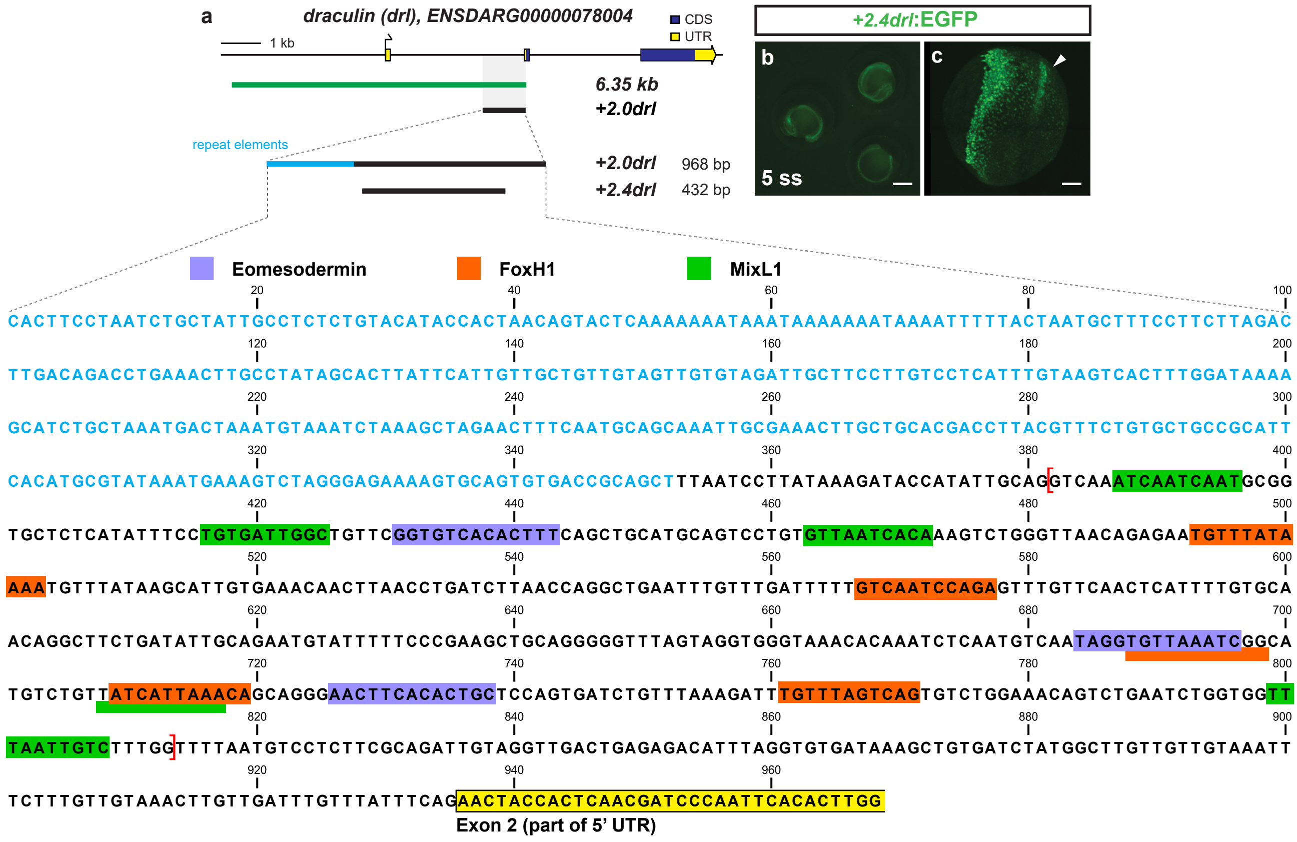

Supplementary Figure 4: Trimming of *+2.0drl* to the minimal 432 bp LPM *+2.4drl* enhancer region

(**a**) Schematic representation of the *-6.35drl* locus including the PCR-based trimming approach to map the smallest LPM specific regulatory enhancer region; repetitive elements highlighted in blue. Sequence depicts the *+2.0drl* region in intron 1 (*Danio rerio* strain Tuebingen chromosome 5, GRCz11 primary assembly, *NC_007116.7:61649227-61650194*): blue text depicts repetitive elements, colored boxes depict predicted transcription factor binding sites (JASPAR database for vertebrates, 80-85% threshold), yellow frame marks the beginning of *drl* exon 2, and red brackets outline the sequence for the *+2.4drl* core sequence. (**b**) Expression of *+2.4drl:EGFP* at 5 ss; arrow indicates axial mesoderm expression, indicating increased promiscuity after trimming from the initial *+2.0drl* element. Scale bar 400 µm (**b**) and 80 µm (**c**).

Supplementary Figure 5

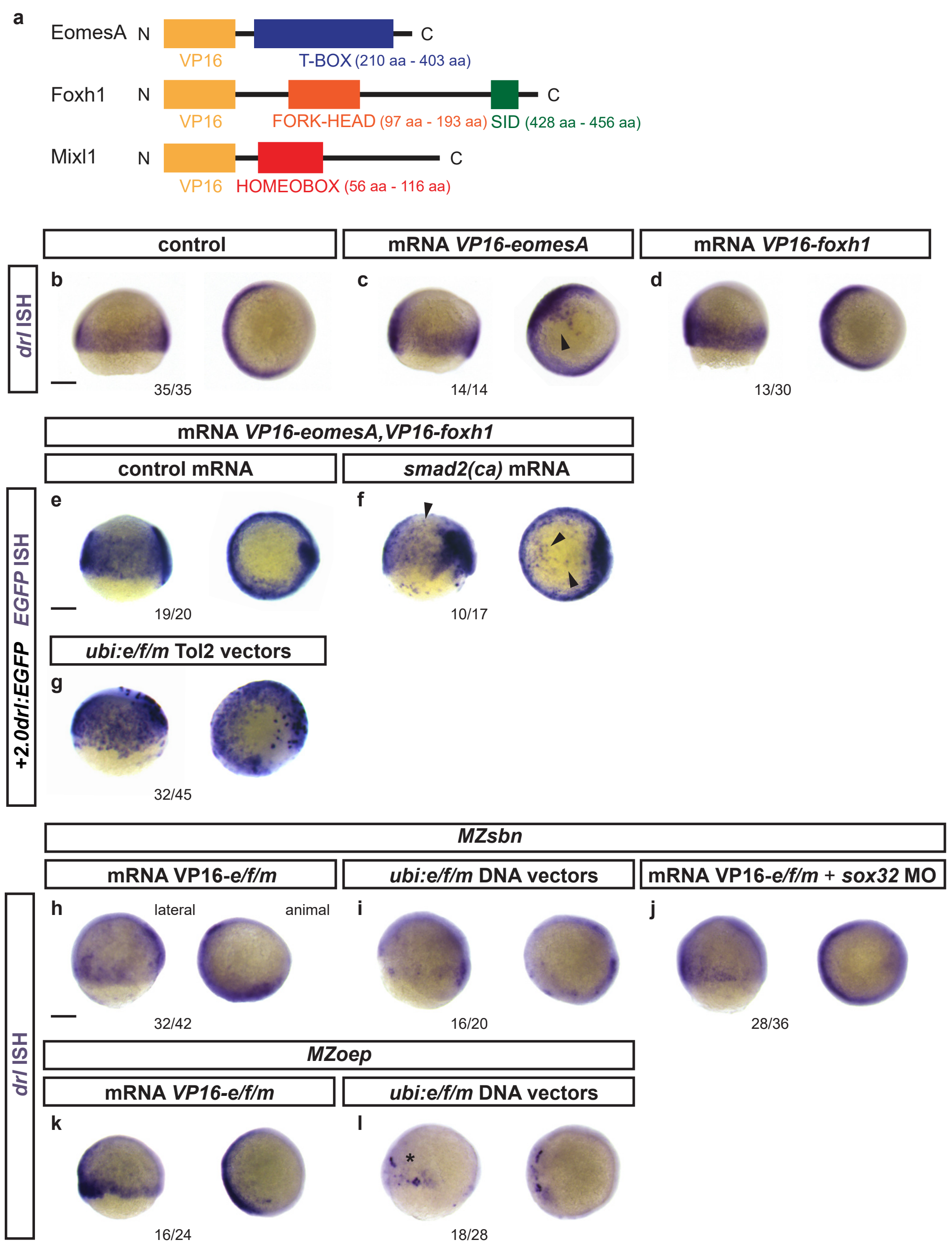

Supplementary Figure 5: Response of endogenous *drl* and *drl* reporters to EomesA, Foxh1, and Mixl1 expression

(**a**) Schematics of EomesA, Foxh1, and Mixl1 fusion proteins with the N-terminally added VP16 transactivation domain to generate constitutively-active transcription factors. Note that the expressed VP16-EomesA is restricted to the T-box of EomesA. (**b-d**) mRNA ISH for endogenous *drl* expression in controls (n=35/35) (**b**) and embryos injected with VP16-fusions based on *eomesA* (n=14/14) (**c**) or *foxh1* (n=13/30) (**d**). (**e,f**) mRNA ISH for *EGFP* transcript expression in *+2.0drl:EGFP* embryos at shield to 70% epiboly stage after injecting a combination of *VP16*-fused *eomesA* and *foxh1* (*e/f*) without (n=19/20) (**e**) or with (n=10/17) (**f**) constitutive-active *smad2* that mimicks pan-Smad signaling (*smad2(ca)*), resulting in dorsal widening of the reporter expression pattern. (**g**) Injection of Tol2-based constructs under ubiquitous *ubi* promoter control to drive native *eomesA* (full-length), *foxh1*, and *mixl1* (*e/f/m*), resulting in mosaic induction of *+2.0drl:EGFP*, including individual dorsal blastomeres (n=32/45). (**h-j**) Endogenous *drl* expression revealed by mRNA ISH in BMP signaling-mutant (*MZsbn*) embryos injected with either (**h**) *VP16-e/f/m* mRNA (n=32/42), (**i**) *ubi:e/f/m* DNA vectors (n=16/20), or (**j**) *VP16-e/f/m* mRNA in an endoderm-perturbed background (*sox32* morpholino) (n=28/36). (**k,l**) mRNA ISH for endogenous *drl* expression in Nodal-mutant (*MZoep*) embryos injected with (**k**) *VP16-e/f/m* mRNA (16/24) or (**l**) *ubi:e/f/m* Tol2 vectors (17/28); note the localized clones of strong *drl* upregulation on the ventral side upon *ubi:e/f/m* injection. Scale bar 250 μm.

Supplementary Figure 6

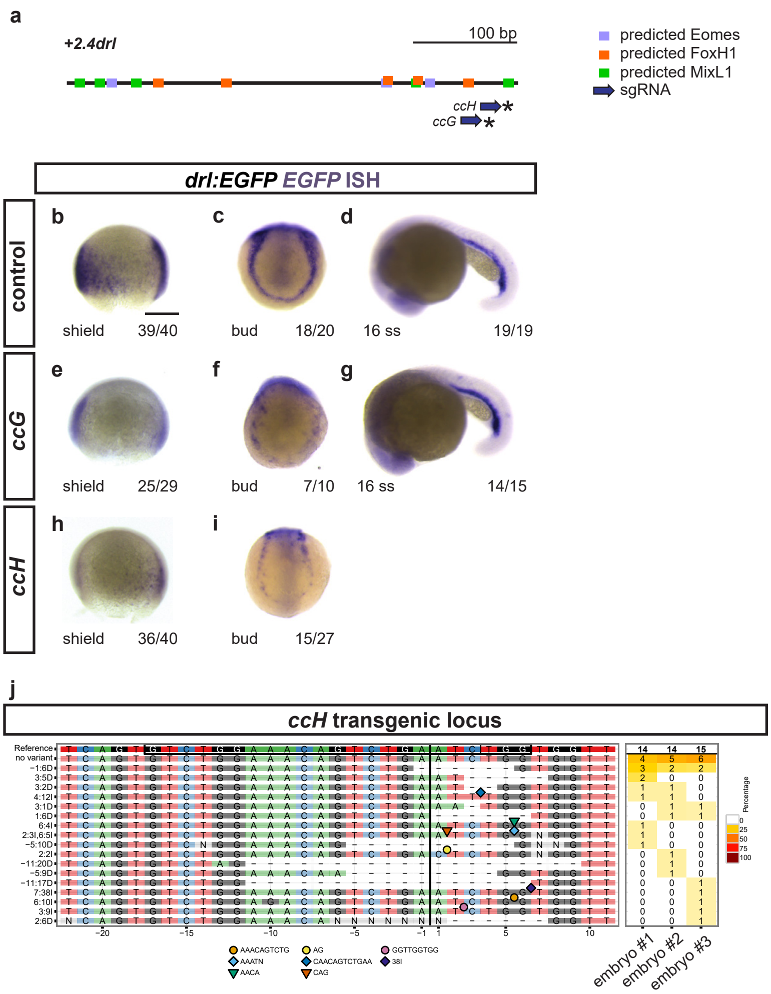

Supplementary Figure 6: Crispant analysis of the *+2.0drl* region

(**a**) Schematic representation of the minimal *+2.4drl* LPM enhancer region (core of *+2.0drl*) with Eomes, FoxH1, and Mixl1 binding site predictions (JASPAR) and sgRNAs *ccG* and *ccH* annotated. (**b-i**) ISH for *EGFP* in *+2.0drl:EGFP* embryos shown for injection controls (**b-d**) or individually mutagenized using reconstituted Cas9 protein-sgRNA complexes with either sgRNA (**e-g**) *ccG* or (**h,i**) *ccH* (asterisks in **a**), both showing mosaic loss or reduction of reporter expression at shield and bud stage compared to uninjected controls. Reporter expression that is dependent on different regulatory elements remains intact upon mutagenesis with both sgRNAs, as observed at 16 ss (**d,g**). These results indicate no overt mutagenesis of essential parts in the transgene itself. (**j**) Panel plot showing mutagenesis efficiency and allele spectrum in three representative *ccH* crispants, as created by CrispRVariants. The genomic reference sequence is shown on top, with the 20 bp *ccH* sgRNA followed by a 3 bp PAM indicated by the boxed regions. The Cas9 cleavage site is represented by a black vertical bar. Deletions are indicated by a minus (-) and insertion sequences by symbols. The right column of the plot shows detected allele frequency per analyzed embryo. Scale bar (**b**) 250 µm.

Supplementary Figure 7

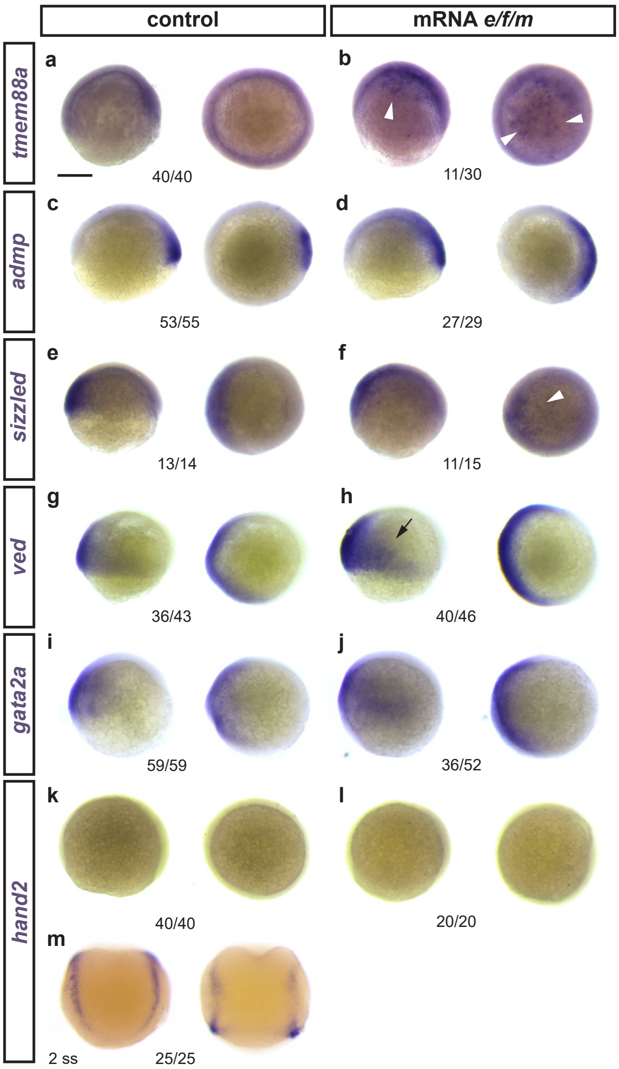

Supplementary Figure 7: Effects of *eomesA, foxh1, and mixl1* misexpression on early developmental genes
(**a-m**) Zebrafish embryos ((**a-l**) shield to 75% epiboly or (**m**) 2 ss) as control or injected with mRNA for *eomesA*, *foxh1*, and *mixl1* (*e/f/m*), with mRNA ISH for indicated candidate genes. Latera view left, animal view right for each condition. (**a**,**b**) The early LPM gene *tmem88a* that is weakly expressed during epiboly (**a**) becomes activated in patches upon e/f/m misexpression (**b**, arrowheads). (**c-j**) Expression of various gastrulation-stage marker genes: *admp* (**c**,**d**), *sizzled* (slightly increased expression ventral, indicated by arrowhead) (**e**,**f**), *ved* (slight increased expression ventral, arrow) (**g**,**h**), *gata2a* (slight ventral expansion) (**i**,**j**). (**k-m**) The somite-stage LPM gene *hand2* does not become ectopically induced upon *e/f/m* misexpression (**k-l**), as tested with a functional mRNA ISH probe (shown for 2 ss in **m**). Indicated n-numbers represent the number of embryos analyzed. Scale bar (**a**) 250 µm.

Supplementary Figure 8

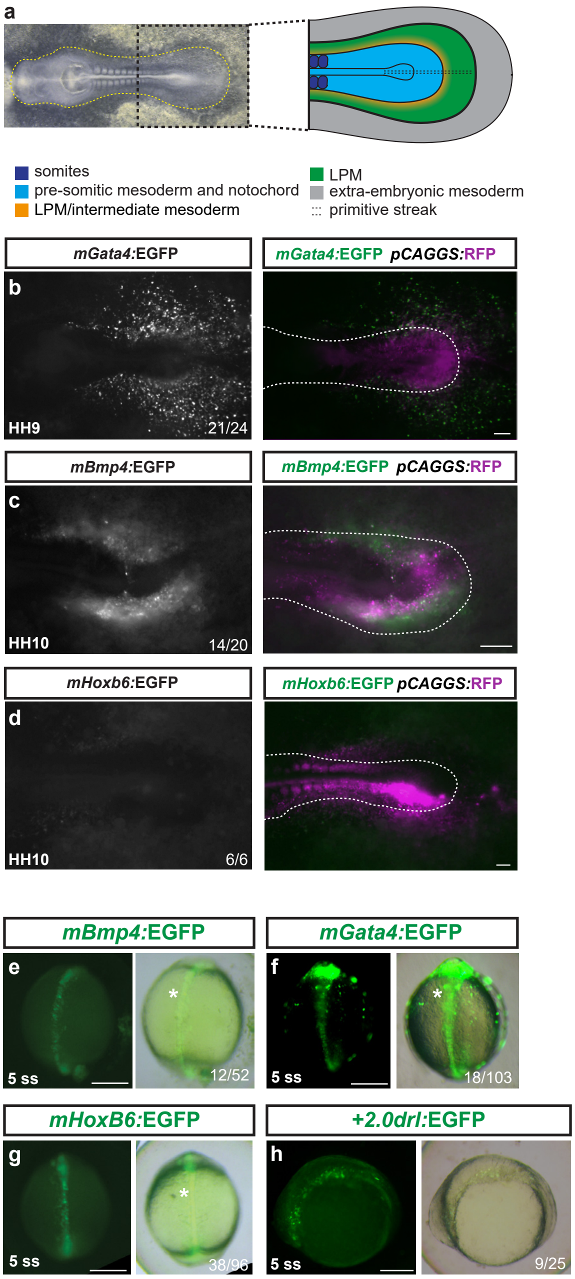

Supplementary Figure 8: Mouse LPM enhancers show specific activity in chick, but not in zebrafish embryos
(**a**) Bright field image of an HH10 stage chicken embryo, anterior to the left, with schematic depiction of the posterior body (right), prospective LPM in green. (**b-d**) *Ex ovo*-cultured chicken embryos at HH9-HH10, electroporated at HH3+/H4 with reporters based on mouse LPM enhancers from *mGata4* (n=21/24) (**b**), *mBmp4* (n=14/20) (**c**), and *mHoxb6* (n=6/6) (**d**) driving *EGFP* (grayscale), including a merged overlay together with electroporation marker plasmid *pCAGGS:RFP* (magenta, driving ubiquitous expression as electroporation control). Dashed lines (**b-d**) indicate the posterior outline of the individual chicken embryos, marking the predicted boundary between embryo and extra-embryonic tissue. (**e-h**) Zebrafish embryos depicted at early somitogenesis stages (dorsal views in **e-g**, lateral view in **h**) injected with EGFP reporters for *mBmp4* (n=12/52) (**e**), *mGata4* (n=18/103) (**f**), and *mHoxb6* (n=38/96) (**g**) compared to *+2.0drl:EGFP* (n=9/25) (**h**), revealing axial mesoderm expression of the tested mouse enhancers. Asterisks point out axial mesoderm expression. Scale bar (**b-d**) 250 µm, (**e-h**) 200 µm.

Supplementary Figure 9

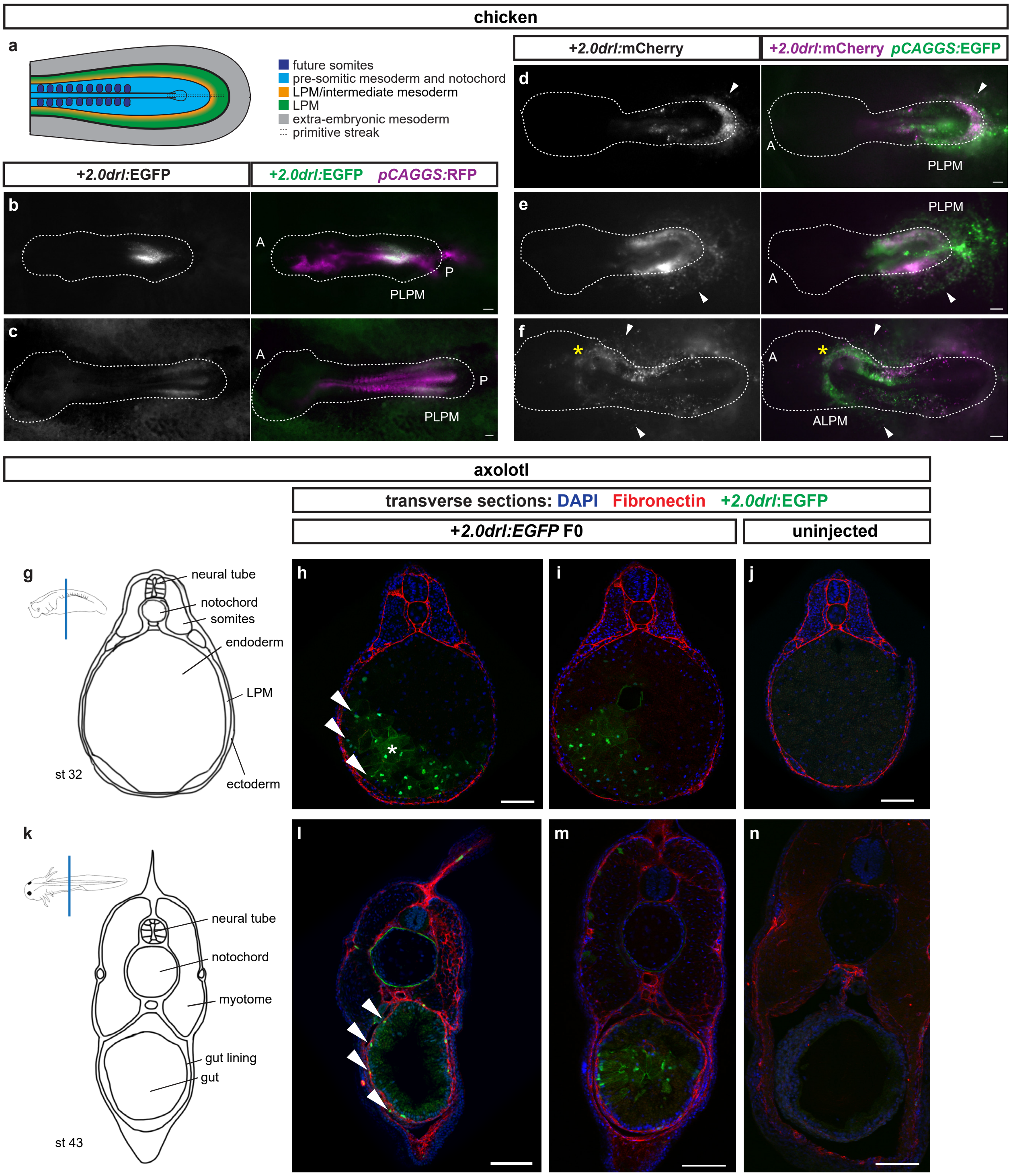

Supplementary Figure 9: Examples of electroporated chicken embryos with *+2.0drl* reporters driving specific expression in the chicken LPM

(**a**) Posterior region of chicken embryo represented in a schematic showing different territories. (**b-f**) *Ex ovo* cultured chicken embryos electroporated with *+2.0drl cis*-regulatory reporters as (**b-c**) *+2.0drl:EGFP*, (**d-f**) *+2.0drl:mCherry* (magenta) together with control plasmid (**b-c**) *pCAGGS:RFP* (magenta) or (**d-g**) *pCAGGS:EGFP* at HH3+/HH4. Arrowheads indicate extra-embryonic endothelial/blood progenitors and asterisks the heart field (**f**). The dashed lines indicate the outline of the chicken embryos based on bright field imaging, with anterior (a) located to the left.

(**g**) Schematic and (**h,i**) confocal Z-stack projections of a transverse section through the trunk region of the transgenic +2.0*drl*:EGFP and (**j**) negative control (uninjected) embryo counterstained with fibronectin antibody at tailbud stage 32, showing mesendoderm (arrowheads) and endodermal EGFP positive cells. (**k**) Schematic and (**l,m**) confocal Z-stack projections of transverse sections through the trunk region of the transgenic +2.0*drl*:EGFP and (**n**) negative control (uninjected) animals at stage 43 showing EGFP positive cells in the gut lining (arrowheads) consistent with LPM origin, and a few cells in the endoderm. Scale bars (**b-f**) 250 μm, (**h-i, l-n**) 200 μm.

Supplementary Figure 10

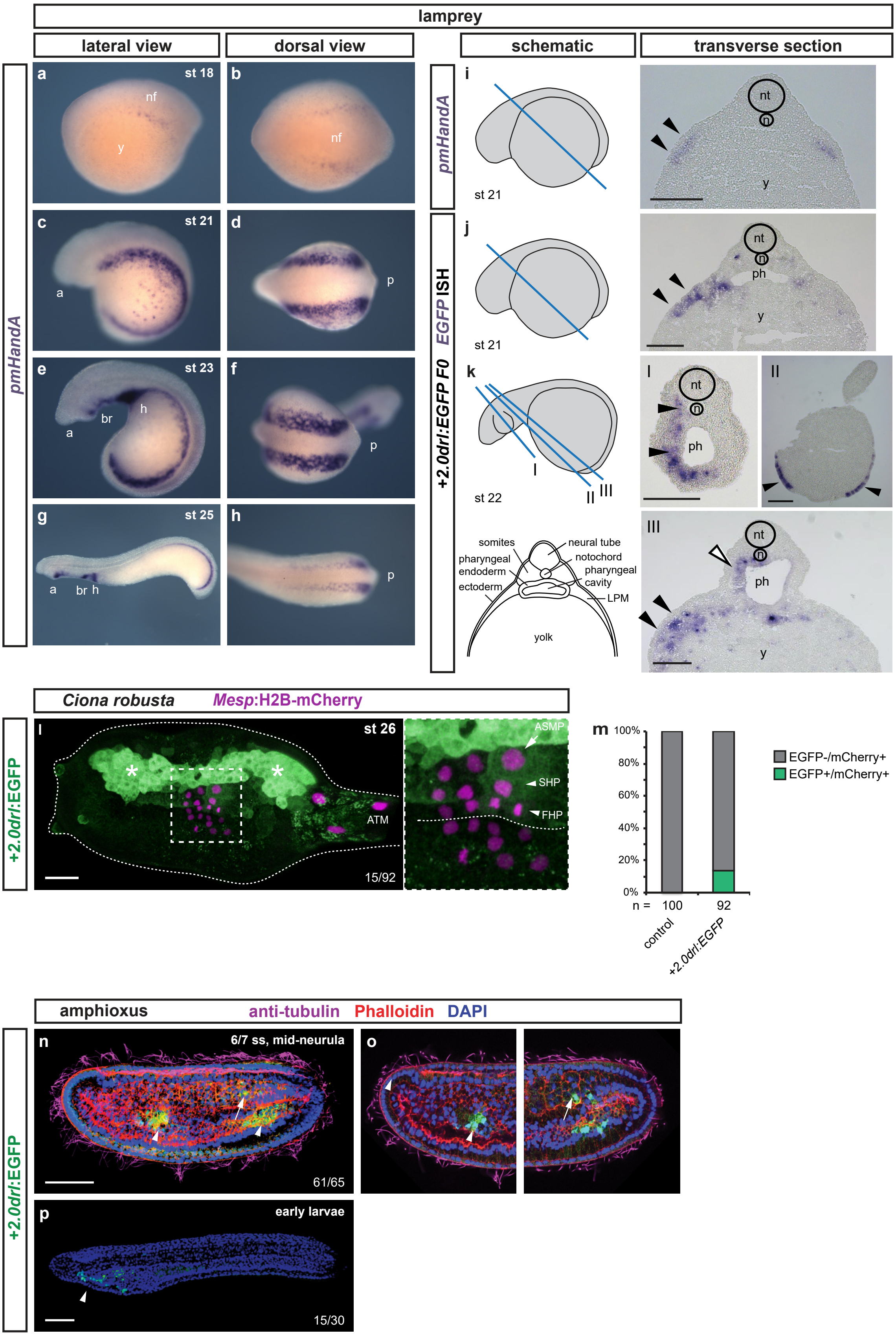

Supplementary Figure 10: *+2.0drl* drives specific reporter expression in lamprey, *Ciona*, and amphioxus

(**a-d**) Whole-mount ISH for *pmHandA* marks the LPM in lamprey embryos from neurula to hatching stages (st 18-25). (**a**) Expression is first detectable as bilateral stripes in the PLPM at st 18. (**b,c**) Later, the PLPM expression expands covering the yolk, and *pmHandA* is upregulated in the heart tube, branchial region, and caudal to the heart. Lateral and dorsal views are shown with the anterior (a) of the embryo to the left. (**i-k**) Transverse sections of fixed st 21-22 embryos. The schematics indicate the plane of sectioning in blue and sections are shown with dorsal to the top. The schematic transverse section represents **k-III**. (**i**) ISH for *pmHandA* at st 21 marking the LPM (black arrowheads). (**j,k**) ISH for *EGFP* in transverse sections of *+2.0drl:EGFP* transient transgenic lamprey embryos fixed at st 21-22, showing enhancer activity in the anterior mesendoderm subjacent to the ectoderm (black arrowheads), in the pharyngeal endoderm (**k-III**, white arrowhead), in the pharyngeal mesoderm (**k**, black arrowheads), and in some embryos in the ectoderm as commonly observed unspecific expression of reporter plasmids (**k-II**, black arrowheads). (**l,m**) Immunostaining for EGFP in *Ciona* larvae embryo (st 26) expressing *drl* reporters (green) and *Mesp*:H2B-mCherry to track the B7.5 nuclei cell lineage (red). (**L**) Expression of *+2.0drl*-driven EGFP reporter in the larvae is stained in the mesenchymal lineage (white asterisks) and in the B7.5 cell progeny including ASM precursors (ASMP) (white arrow) and both cardiac first and second heart precursors (FHPs and SHPs) (white arrowheads), zoomed in box. Anterior to the left. (**m**) Proportion of larvae embryos expressing both GFP and mCherry in the B7.5 lineage when co-electroporated *drl* reporter and *Mesp*:H2B-mCherry in comparison to the control. N = number of electroporated larval halves. (**n-p**) Confocal Z-stack of amphioxus embryo at mid-neurula stage (6/7 ss), injected with *+2.0drl:EGFP*, showing specific reporter activity in the lateral endoderm (arrowhead), and ventral half of the somites (n=61/65, arrows). Embryos counterstained with Phalloidin (red), embryos/larvae with DAPI (blue), and anti-acetylated tubulin antibody (magenta). Lateral view shown as 3D-rendering (**n**) or Z-stack sagittal sections (**o**)**,** anterior to the left and dorsal to the top. At early larvae stage (**p**), the activity of *+2.0drl* reporter was observed in the developing pharynx (n=15/30, arrowhead). Branchial region (br), heart (h), neural folds (nf), neural tube (nt), notochord (n), pharynx (ph), and yolk (y) labeled (**i-k**). Scale bars (**i-k**) 200 μm, (**l**) 25 μm, (**n,p**) 50 μm.

Supplementary Methods

Transgene cloning

The transgene vectors *-1.0drl:EGFP (pDestTol2pA2_-1.0drl:EGFP)* and *[proximal]drl:EGFP (pDestTol2pA2_[proximal]drl:EGFP)* were assembled from pENTR/5'_*-1.0drl* or pENTR/5'_*[proximal]drl* together with Tol2kit vectors *#383* (*pME-EGFP*), *#302* (*p3E_SV40polyA*), and *#394* (*pDestTol2A2*) as backbone. *+2.0drl:EGFP (pDestTol2pA2_+2.0drl:EGFP)* and *+2.4drl:EGFP (pDestTol2pA2_ +2.4drl:EGFP)* were assembled from pENTR/5'_*+2.0drl* or pENTR/5'_*+2.4drl* together with *pME-β-globin_minpromoter_EGFP*[1], Tol2kit vectors *#302* (*p3E_SV40polyA*), and *#394* (*pDestTol2A2*) as backbone[2]. *+2.0drl:creERT2 (pDestTol2CY_+2.0drl:creERT2,alpha-crystallin:YFP)* and *-5.0sox17:creERT2 (pDestTol2CY_-5.0sox17:creERT2,alpha-crystallin:YFP)* were assembled from *pENTR/5'_+2.0drl* or *pENTR/5'_-5.0sox17* together with *pCM293* (*pENTR/D_creERT2*)[3], Tol2kit vector *#302* (*p3E_SV40polyA*), and *pCM326 (pDestTol2CY)* as backbone[4]. Genomic coordinates for the *+2.0drl* enhancer used in the described constructs are *chr5:61,649,227-61,650,194* (*GRCz11/danRer11*). Transcription factor binding sites were predicted using the JASPAR online interface[5]. Synteny analysis was performed using the Genomicus web server[6].

The regulatory elements of mouse-specific LPM enhancers were PCR-amplified (Supplementary Table 1; regulatory elements), TOPO-cloned into the *pENTR5'* plasmid, and assembled together with *pKD001* (*pME-β-globin_minpromoter_EGFP* with improved Kozak sequence), Tol2kit vectors *#302* (*p3E_SV40polyA*), and *#394* (*pDestTol2A2*) as backbone.

Assembled reporter constructs were injected at a concentration of 25 ng/μl together with 25 ng/μl *Tol2* mRNA for Tol2-mediated zebrafish transgenesis. Injected F0 founders were screened for specific EGFP or *alpha-crystallin:*YFP expression. Zebrafish were raised to adulthood and screened in F1 for germline transmission. Single-insertion transgenic strains were generated, and microscopy images were taken on a Leica M205FA with a Leica DFC450C digital camera or a Leica SP8 confocal microscope with Plan-Apochromat 20x/0.5 objective. Images were processed using Leica LAS and Fiji[7].

Zebrafish strains

Established transgenic and mutant lines used in this study included *drl:EGFP*[4]*, drl:mCherry^{zh705}* (Ref. 8), *drl:creERT2*[4], *ubi:creERT2*[3], *ubi:switch*[3], *hsp70l:Switch^{zh701}* (Ref. 9), *lmo2:dsRED2*[10]*, scl/tal1:EGFP*[11]*, pax2.1:EGFP*[12]*, hand2:EGFP*[13], *actb2:h2afva-mCherry*[14], maternal and zygotic EGF-CFC co-receptor *oep* gene mutants (*MZoep*)[15], and maternal-zygotic *somitabun^{dtc24}* mutants (*sbn,* dominant-negative *smad5*)[16,17].

Zebrafish CreERT2-based lineage tracing

Lineage tracing experiments were performed by crossing female *hsp70l:Switch*[9] or *ubi:Switch*[3] reporter carriers with the male *creERT2* drivers *drl:creERT2*[4], *sox17:creERT2*, and *+2.0drl:creERT2*. Embryos were induced using 4-Hydroxytamoxifen (4-OHT) (H7904; Sigma H7904) from fresh or pre-heated (65°C for 10 min) stock solutions in DMSO at a final concentration of 10 μM in E3 embryo medium at indicated time points. Embryos were washed in untreated E3 medium at 24 hpf. To induce EGFP transcription in *hsp70l:Switch* carrying embryos, the embryos were incubated at 37°C for 1 hour, 2-3 hours before fixation. Embryos were fixed with 4% paraformaldehyde (PFA) overnight at 4°C at 3 dpf and processed for confocal analysis.

Zebrafish morpholino and crispant experiments

The previously established and validated *sox32* morpholino[18] was synthesized by GeneTools (sequence: *5'-TGCTTTGTCATGCTGATGTAGTGGG-3'*) and dissolved in nuclease-free water to a stock concentration of 10 μg/μl. The *sox32* morpholino injection mix of 4 μg/μl was incubated for 10 min at 65°C, and 8 ng were microinjected into one-cell stage zebrafish embryos.

sgRNAs were obtained in an oligo-based approach[19]. Primer extension was performed using Phusion polymerase (NEB) of forward primer 5'-*GAAATTAATACGACTCACTATAGG-N_{20}-GTTTTAGAGCTAGAAATAGC*-3' (including a 20 nt target site) and invariant reverse primer 5'-*AAAAGCACCGACTCGGTGCCACTTTTTCAAGTTGATAACGGACTAGCCTTATTTTAACTTGCTATTTCTAGCTCTAAAAC*-3' (PAGE-purified). The following target sites are shown in this manuscript: 5'-

AGATTTGTTTAGTCAGTGTC-3' (*drl ccG*), 5'-*GTCTGGAAACAGTCTGAATC*-3' (*drl ccH*), 5'-*GAGACTTCGCCCTTCGGTTC*-3' (*mixl1 ccB*), and 5'-*GACAGAACAGGCCACGTTGA*-3' (*mezzo ccA*).

In vitro transcription of sgRNAs was performed using MAXIscript T7 (Ambion). Afterwards, RNA was precipitated with ammonium acetate, washed with 75% ethanol, and dissolved in DEPC water. RNA quality was checked on a MOPS gel. RNPs of Cas9 protein (Cas9-mCherry, *pMJ293*[19]; available from Addgene) and sgRNA were *in vitro*-assembled for 10 min at 37°C in 300 mM KCl to ensure maximum cleavage efficiency and microinjected into the cell of one-cell stage embryos[19]. The CRISPR target regions in the transgenic locus were amplified using specific target region amplification primers (Supplementary Table 1; target region amplifications).

Sequencing analysis was performed with the R software package CrispRVariants for allele- and sequence-level variant counting and visualization[20].

Zebrafish overexpression experiments

For early developmental transcription factor genes, ORFs were PCR-amplified from mixed-stage zebrafish cDNA using ORF-specific primers (Supplementary Table 1; coding sequence primers); full-length zebrafish *eomesA* was derived from I.M.A.G.E clone *IRBOp991A0739D* (Source BioScience LifeSciences, UK). All CDS were TOPO-cloned into the *pENTR/D*™ Directional TOPO® plasmid (Invitrogen) according to the manufacturer's instructions. Constitutive-active Smad2, which encodes an N-terminal truncation of zebrafish Smad2[21], was amplified from zebrafish cDNA and subcloned into *pENTR1A* (*pCM269*). Subsequent cloning reactions were performed with the Multisite Gateway system with LR Clonase II Plus (Invitrogen) according to the manufacturer's instructions. The *CDS* as pENTR/D vectors (*pENTR1A* for *smad2(ca)*) were assembled with *pENTR5'_ubi*[3]*, pENTR5'_T7-VP16*, *pENTR5'_T7-eng* or *pENTR5'_T7* together with Tol2kit vectors *#302* (*p3E_SV40polyA*), and *#394* (*pDestTol2A2*) as backbone[2]. Afterwards, plasmids were linearized (in case of *T7-VP16, T7-eng*, or *T7*) and *in vitro* transcribed (Roche). The *EomesA-VP16* plasmid (containing 153aa-431aa of the zebrafish EomesA ORF)[22] was kindly provided by Dr. Rebecca Burdine. The plasmid was linearized via NotI followed by SP6 *in vitro* transcription. All mRNAs were precipitated with ammonium acetate, washed with 75% ethanol, and dissolved in DEPC water. mRNA quality was checked on MOPS gels.

Supplementary Table 1: Primer sequences

Regulatory elements

-1.0drl zebrafish	*5’-TTTCAATTTCAAAGGAGC-3’*<br>*5’-TGTTTTGAGTGAGTCGGTTAGG-3’*
[proximal]drl zebrafish	*5’- CTGTCATCATTCACTCACCC-3’*<br>*5’-CAACTGGGACATATTTAAACC-3’*
+2.0drl zebrafish	*5’-CACTTCCTAATCTGCTATTGCC-3’*<br>*5’-CCAAGTGTGAATTGGGATCG-3’*
+2.0drl<br>*for lamprey expression*	*5’-CCCTCGAGGTCGACGCACTTCCTAATCTGCTATTG-3’*<br>*5’- GAGGATATCGAGCTCGCCAAGTGTGAATTGGGATC-3’*
+2.0drl<br>*for Ciona expression*	*5’-CTCGAGCACTTCCTAATCTGCTATTGCC-3’ (w/ Xho1)*<br>*5’-TCTAGACCAAGTGTGAATTGGGATCG-3’ (w/ Xba1)*
+2.4drl:EGFP zebrafish	*5’-GTCAAATCAATCAATGCGGTGC-3’*<br>*5’-CCAAAGACAATTAAACCACC-3’*
+2.4drl:EGFP<br>*for lamprey expression*	*5’-AGGGTAATGAGGGCCGTCAAATCAATCAATGCGGTGCTC-3’*<br>*5’GAGGATATCGAGCTCGCCAAAGACAATTAAACCACCAGATTCAG-3’*
+2.4drl:EGFP<br>*for Ciona expression*	*5’-CTCGAGGTCAAATCAATCAATGCGG-3’ (w/ Xho1)*<br>*5’-TCTAGACCAAAGACAATTAAACCACC-3’ (w/ Xba1)*
-5.0sox17	*5’-TGACGACCCCCAGGTCAACC-3’*<br>*5’-CTCAAACACGCACCGGGC-3’*
mBmp4	*5’-GGGGATGAAAGTAGCATCCTG-3’*<br>*5’-TTCCACTTTGCTTCCCAAACTGG-3’*
mHoxb6	*5’-AATGGAGAACTGGCTGGTG-3*<br>*5’-ATCCTTTAGAGCAGCAACCC-3’*
mGata4	*5’-CCTTCCTATACACGCTTGC-3’*<br>*5’-TCCTTCCCAAGCCCTGAGG-3’*

Target region amplification

drl	*5’-AATCACAAAGTCTGGGTTAACAGAG-3’*<br>*5’-CGACCAGGATGGGCACCAC-3’ (EGFP region)*
mixl1	*5’-GGCTGCTGTTATCAGTCTC-3’*<br>*5’-GGGATGGTGGTTGGGTCG-3’*
mezzo	*5’-CGCTGTGGCGACCTCTGA-3’*<br>*5’-CTTGCTCTCTGGAAGCAGCG-3’*

**Coding sequences**	
foxh1	*5’-CACCATGACAAAGCACTGGGGG-3’*<br>*5’-TTAAAGAGAATATTTGCAAAGG-3’*
mixl1	*5’-CACCATGGCAGTCGTGCACGGAAACC-3’*<br>*5’-CTAAATGAAGCCATTAAAACC-3’*

***in situ* hybridization**	
EGFP	*5’-ATGGTGAGCAAGGGCGAGGAGC-3’*<br>*5’-TAATACGACTCACTATAGGG-3’*
drl	*5’-ATGAAGAATACAACAAAACCC-3’*<br>*5’-TAATACGACTCACTATAGGTGAGAAGCTCTGGCCGC-3’*
hand2	*5’-CACCATGAGTTTAGTTGGAGGGTTTCC-3’*<br>*5’-TAATACGACTCACTATAGGGTCATTGCTTCAGTTCCAATGCC-3’*
foxf1	*5’-ATACCGCGCTATCATACC-3’*<br>*5’-GAATTCTAATACGACTCACTATAGGGAAAGCAGTCTTAACTCTGG-3’*
ved	*5’-CACCATGAAGGGTCAGTTCTCC-3’*<br>*5’-TAATACGACTCACTATAGGGCACAATAAAGCCAGTCC-3’*
tmem88a	*5’-ATGAGTCTTCCACGAAACGG-3’*<br>*5’-TAATACGACTCACTATAGGGATGAAGCACAGGCCC-3’*

Supplementary References

1. Tamplin, O. J. *et al.* Hematopoietic stem cell arrival triggers dynamic remodeling of the perivascular niche. *Cell* **160**, 241–52 (2015).
2. Kwan, K. M. *et al.* The Tol2kit: a multisite gateway-based construction kit for Tol2 transposon transgenesis constructs. *Dev Dyn* **236**, 3088–3099 (2007).
3. Mosimann, C. *et al.* Ubiquitous transgene expression and Cre-based recombination driven by the ubiquitin promoter in zebrafish. *Development* **138**, 169–177 (2011).
4. Mosimann, C. *et al.* Chamber identity programs drive early functional partitioning of the heart. *Nat. Commun.* **6**, 8146 (2015).
5. Khan, A. *et al.* JASPAR 2018: update of the open-access database of transcription factor binding profiles and its web framework. *Nucleic Acids Res.* **46**, D260–D266 (2018).
6. Nguyen, N. T. T., Vincens, P., Roest Crollius, H. & Louis, A. Genomicus 2018: karyotype evolutionary trees and on-the-fly synteny computing. *Nucleic Acids Res.* **46**, D816–D822 (2018).
7. Schindelin, J. *et al.* Fiji: an open-source platform for biological-image analysis. *Nat. Methods* **9**, 676–82 (2012).
8. Sánchez-Iranzo, H. *et al.* Tbx5a lineage tracing shows cardiomyocyte plasticity during zebrafish heart regeneration. *Nat. Commun.* **9**, 428 (2018).
9. Felker, A. *et al.* Continuous addition of progenitors forms the cardiac ventricle in zebrafish. *Nat. Commun.* **9**, 2001 (2018).
10. Zhu, H. *et al.* Regulation of the lmo2 promoter during hematopoietic and vascular development in zebrafish. *Dev Biol* **281**, 256–269 (2005).
11. Jin, H. *et al.* The 5’ zebrafish scl promoter targets transcription to the brain, spinal cord, and hematopoietic and endothelial progenitors. *Dev Dyn* **235**, 60–67 (2006).
12. Picker, A., Scholpp, S., Böhli, H., Takeda, H. & Brand, M. A novel positive transcriptional feedback loop in midbrain-hindbrain boundary development is revealed through analysis of the zebrafish pax2.1 promoter in transgenic lines. *Development* **129**, 3227–39 (2002).
13. Yin, C., Kikuchi, K., Hochgreb, T., Poss, K. D. & Stainier, D. Y. R. Hand2 regulates extracellular matrix remodeling essential for gut-looping morphogenesis in zebrafish. *Dev. Cell* **18**, 973–984 (2010).
14. Krens, S. F. G., Möllmert, S. & Heisenberg, C.-P. Enveloping cell-layer differentiation at the surface of zebrafish germ-layer tissue explants. *Proc. Natl. Acad. Sci. U. S. A.* **108**, E9-10; author reply E11 (2011).
15. Gritsman, K. *et al.* The EGF-CFC Protein One-Eyed Pinhead Is Essential for Nodal Signaling. *Cell* **97**, 121–132 (1999).
16. Mullins, M. C. *et al.* Genes establishing dorsoventral pattern formation in the zebrafish embryo: the ventral specifying genes. *Development* **123**, 81–93 (1996).
17. Hild, M. *et al.* The smad5 mutation somitabun blocks Bmp2b signaling during early dorsoventral patterning of the zebrafish embryo. *Development* **126**, 2149–59 (1999).
18. Sakaguchi, T., Kuroiwa, A. & Takeda, H. A novel sox gene, 226D7, acts downstream of Nodal signaling to specify endoderm precursors in zebrafish. *Mech. Dev.* **107**, 25–38 (2001).
19. Burger, A. *et al.* Maximizing mutagenesis with solubilized CRISPR-Cas9 ribonucleoprotein complexes. *Development* **143**, 2025–37 (2016).
20. Lindsay, H. *et al.* CrispRVariants charts the mutation spectrum of genome engineering experiments. *Nat. Biotechnol.* **34**, 701–702 (2016).
21. Dick, A., Mayr, T., Bauer, H., Meier, A. & Hammerschmidt, M. Cloning and characterization of zebrafish smad2, smad3 and smad4. *Gene* **246**, 69–80 (2000).
22. Bruce, A. E. E. *et al.* The maternally expressed zebrafish T-box gene eomesodermin regulates organizer formation. *Development* **130**, 5503–5517 (2003).
