# Supplementary material for: A conserved regulatory program initiates lateral plate mesoderm emergence across chordates
Source: Nat Commun. 2019 Aug 26;10:3857. doi: 10.1038/s41467-019-11561-7 (PMC6710290; doi:10.1038/s41467-019-11561-7)
Supplement: Supplementary file 7 — Description of Additional Supplementary Files [file 41467_2019_11561_MOESM7_ESM.pdf]

Legends for Supplementary Movies:

Title: Supplementary Movie 1: 3D lightsheet imaging of the arising *drl* reporter-labeled LPM
Description: SPIM time lapse imaging of a zebrafish embryo transgenic for *drl*:EGFP (green) together with the nuclear marker *actb2:*H2afva-mCherry (magenta) to visualize LPM emergence from 50% epiboly to 10 ss.

Title: Supplementary Movie 2: Panoramic lightsheet imaging of the arising *drl* reporter-labeled LPM
Description: Mercator projection of SPIM time lapse imaging of zebrafish embryos transgenic for *drl:EGFP* to visualize LPM emergence from 50% epiboly to 10 ss. From somitogenesis onwards, the embryo is oriented with anterior to the left and posterior to the right.

Title: Supplementary Movie 3: 3D lightsheet imaging showing overlap between *drl* reporter expression and endoderm during gastrulation
Description: SPIM time lapse imaging of *drl:mCherry* (red) combined with *sox17:EGFP* (green) to visualize the *drl*-expressing emerging cell population versus endoderm from 50% epiboly until 16 ss.

Title: Supplementary Movie 4: Panoramic lightsheet imaging showing overlap between *drl* reporter expression and endoderm during gastrulation
Description: Mercator projection of SPIM time lapse imaging of *drl:mCherry* (magenta) combined with *sox17:EGFP* to visualize the *drl*-expressing emerging cell population versus endoderm from 50% epiboly until 16 ss. From somitogenesis onwards, the embryo is oriented with anterior to the left and posterior to the right. Double-positive cells during gastrulation and early somitogenesis are shown in blue.
