# Supplementary material for: A conserved regulatory program initiates lateral plate mesoderm emergence across chordates
Source: Nat Commun. 2019 Aug 26;10:3857. doi: 10.1038/s41467-019-11561-7 (PMC6710290; doi:10.1038/s41467-019-11561-7)
Supplement: Supplementary file 8 — Reporting Summary [file 41467_2019_11561_MOESM8_ESM.pdf]

nature research

Corresponding author(s): Dr. Christian Mosimann

Last updated by author(s): YYYY-MM-DD

Reporting Summary

Nature Research wishes to improve the reproducibility of the work that we publish. This form provides structure for consistency and transparency in reporting. For further information on Nature Research policies, see Authors & Referees and the Editorial Policy Checklist.

Statistics

For all statistical analyses, confirm that the following items are present in the figure legend, table legend, main text, or Methods section.

n/a	Confirmed	
☐	☒	The exact sample size (*n*) for each experimental group/condition, given as a discrete number and unit of measurement
☐	☒	A statement on whether measurements were taken from distinct samples or whether the same sample was measured repeatedly
☒	☐	The statistical test(s) used AND whether they are one- or two-sided<br>*Only common tests should be described solely by name; describe more complex techniques in the Methods section.*
☒	☐	A description of all covariates tested
☒	☐	A description of any assumptions or corrections, such as tests of normality and adjustment for multiple comparisons
☒	☐	A full description of the statistical parameters including central tendency (e.g. means) or other basic estimates (e.g. regression coefficient) AND variation (e.g. standard deviation) or associated estimates of uncertainty (e.g. confidence intervals)
☒	☐	For null hypothesis testing, the test statistic (e.g. F, t, r) with confidence intervals, effect sizes, degrees of freedom and P value noted<br>*Give P values as exact values whenever suitable.*
☒	☐	For Bayesian analysis, information on the choice of priors and Markov chain Monte Carlo settings
☒	☐	For hierarchical and complex designs, identification of the appropriate level for tests and full reporting of outcomes
☒	☐	Estimates of effect sizes (e.g. Cohen's d, Pearson's r), indicating how they were calculated

Our web collection on statistics for biologists contains articles on many of the points above.

Software and code

Policy information about availability of computer code

Data collection	No software was used.
Data analysis	Microsoft Excel (commercial); ImageJ/Fiji (freeware)

For manuscripts utilizing custom algorithms or software that are central to the research but not yet described in published literature, software must be made available to editors/reviewers. We strongly encourage code deposition in a community repository (e.g. GitHub). See the Nature Research guidelines for submitting code & software for further information.

Data

Policy information about availability of data

All manuscripts must include a data availability statement. This statement should provide the following information, where applicable:

- Accession codes, unique identifiers, or web links for publicly available datasets
- A list of figures that have associated raw data
- A description of any restrictions on data availability

The authors declare that the data supporting the findings of this study are available within the paper and its supplementary information files. Reagents are available upon request.

Field-specific reporting

Please select the one below that is the best fit for your research. If you are not sure, read the appropriate sections before making your selection.

☒ Life sciences ☐ Behavioural & social sciences ☐ Ecological, evolutionary & environmental sciences

Life sciences study design

All studies must disclose on these points even when the disclosure is negative.

Sample size	Sample sizes were chosen based on clutch sizes of embryos per used model organism. Injection-based assays and stainings were performed to reach effect sizes of >10 of the observed effect in all combined experiments of one type.
Data exclusions	No data was excluded.
Replication	Injections and stainings were done in duplicate or triplicate cohorts depending on embryo availability per model organism to ensure replication. See also Sample size above.
Randomization	No randomization was performed, as individual experiments were performed with either injected effectors, chemical compounds of known effect, or based on genetic criteria. Each experiment contained the appropriate negative control. Downstream stainings and analysis was performed with the same criteria and parameters.
Blinding	Blinding is was not applied as experimental results are qualitative, not quantitative with the performed genetic and molecular experiments.

Reporting for specific materials, systems and methods

We require information from authors about some types of materials, experimental systems and methods used in many studies. Here, indicate whether each material, system or method listed is relevant to your study. If you are not sure if a list item applies to your research, read the appropriate section before selecting a response.

Materials & experimental systems

n/a	Involved in the study	
☐	☒	Antibodies
☒	☐	Eukaryotic cell lines
☒	☐	Palaeontology
☐	☒	Animals and other organisms
☒	☐	Human research participants
☒	☐	Clinical data

Methods

n/a	Involved in the study	
☒	☐	ChIP-seq
☒	☐	Flow cytometry
☒	☐	MRI-based neuroimaging

Antibodies

Antibodies used	anti-Fibronectin antibody (ab6328, Abcam); anti-GFP (1:500, mouse mAb, Roche); anti-acetylated tubulin (T6793 Sigma-Aldrich, clone 6-11B-1, mouse ascites, 1:500)
Validation	* anti-Fibronectin antibody; a standard reagent used in axolotls (see 10.1371/journal.pone.0032875 as example).<br>* anti-GFP (1:500, mouse mAb, Roche); a standard antibody that is widely used to detect GFP (see 10.1038/ncomms5830 as example for use in Ciona).<br>* anti-acetylated tubulin (AcTub) is a standard in the amphioxus field (see 10.1073/pnas.1100045109 as example).

Animals and other organisms

Policy information about studies involving animals; ARRIVE guidelines recommended for reporting animal research

Laboratory animals	* Zebrafish embryos (AB, TU strains, and transgenics of mixed background).<br>* Lamprey embryos from lab-kept wild catch<br>* Chicken embryos from commercially purchased, fertilized eggs (Switzerland)<br>* Ciona robusta embryos, derived from commercially obtained adults<br>* Axolotl embryos, from White mutant (d/d) adults.<br>* Amphioxus embryos, derived from mating of wild-caught adults (France)
Wild animals	This study did not use wild-caught animals for data collection.
Field-collected samples	This study did not use field-collected samples for data collection.
Ethics oversight	* Zebrafish and chick experiments were carried out in accordance with the recommendations of the national authorities of Switzerland (Animal Protection Ordinance). The protocols and the experiments were approved by the cantonal veterinary office of the Canton Zurich (Kantonales Veterinäramt, permit no. 150). Zebrafish care and all experimental procedures were carried out in accordance with the European Communities Council Directive (86/609/EEC), according to which all embryo experiments performed before 120 hours post fertilization are not considered animal experimentation and do not require ethics approval.

Adult zebrafish for breeding were kept and handled according to animal care regulation of the Kantonales Veterinäramt Zürich (TV4209).

* White mutant (d/d) axolotls (Ambystoma mexicanum) were obtained from the axolotl facility at the TUD-CRTD Center for Regenerative Therapies Dresden, Germany.

* Lamprey studies were conducted in accordance with the Guide for the Care and Use of Laboratory Animals of the National Institutes of Health, and protocols were approved by the Institutional Animal Care and Use Committees of the California Institute of Technology (Protocol # 1436-11).

* For chick, Ciona, and amphioxus experiments at the described stages, no ethics approval is required by Swiss Law (experiments before stage 26) or no ethics regulations are in place due to the non-vertebrate character of the involved species (Ciona, amphioxus).

Note that full information on the approval of the study protocol must also be provided in the manuscript.
